# Transcriptomic, Proteomic, and Functional Assays Underline the Dual Role of Extrapallial Hemocytes in Immunity and Biomineralization in the Hard Clam *Mercenaria mercenaria*

**DOI:** 10.3389/fimmu.2022.838530

**Published:** 2022-02-22

**Authors:** Caroline Schwaner, Sarah Farhat, John Haley, Emmanuelle Pales Espinosa, Bassem Allam

**Affiliations:** ^1^ School of Marine and Atmospheric Sciences, Stony Brook University, Stony Brook, NY, United States; ^2^ Stony Brook University Biological Mass Spectrometry Center, Stony Brook Medicine, Stony Brook, NY, United States

**Keywords:** clams, biomineralization, immunity, extrapallial fluid, hemolymph, hemocytes

## Abstract

Circulating hemocytes in the hemolymph represent the backbone of innate immunity in bivalves. Hemocytes are also found in the extrapallial fluid (EPF), the space delimited between the shell and the mantle, which is the site of shell biomineralization. This study investigated the transcriptome, proteome, and function of EPF and hemolymph in the hard clam *Mercenaria mercenaria*. Total and differential hemocyte counts were similar between EPF and hemolymph. Overexpressed genes in the EPF were found to have domains previously identified as being part of the “biomineralization toolkit” and involved in bivalve shell formation. Biomineralization related genes included chitin-metabolism genes, carbonic anhydrase, perlucin, and insoluble shell matrix protein genes. Overexpressed genes in the EPF encoded proteins present at higher abundances in the EPF proteome, specifically those related to shell formation such as carbonic anhydrase and insoluble shell matrix proteins. Genes coding for bicarbonate and ion transporters were also overexpressed, suggesting that EPF hemocytes are involved in regulating the availability of ions critical for biomineralization. Functional assays also showed that Ca^2+^ content of hemocytes in the EPF were significantly higher than those in hemolymph, supporting the idea that hemocytes serve as a source of Ca^2+^ during biomineralization. Overexpressed genes and proteins also contained domains such as C1q that have dual functions in biomineralization and immune response. The percent of phagocytic granulocytes was not significantly different between EPF and hemolymph. Together, these findings suggest that hemocytes in EPF play a central role in both biomineralization and immunity.

## Introduction

Bivalves have an open circulatory system populated by hemocytes, the quintessential component of bivalve immunity. Hemocytes function in several additional physiological processes including wound healing, biomineralization, nutrition, and transport (1–4). Hemocytes in the hemolymph, the blood of bivalves, are well studied and known to serve as the main constituent of innate immunity (3, 5–7). Hemocytes release a broad range of proteins including immune effectors into the plasma of hemolymph, contributing to humoral immunity of bivalves (1, 6, 8). Hemocytes are also found in the extrapallial fluid (EPF), an aqueous microenvironment between the mantle and the shell which is the site of shell formation. While hemocytes are believed to play roles in biomineralization (4, 9), there is much less known about functional differences between cells present in EPF and those in the circulatory system.

Differences between populations of hemocytes have primarily focused on morphological diversity and classification into the subpopulations granulocytes and agranulocytes (10–12). These classifications are based on size and presence or absence of granules. While it is known that granulocytes are more phagocytic than agranulocytes (13), functional differentiation of hemocyte populations, and specifically differentiation between hemocytes from different fluids, lags behind. The advent of -omics technologies enable determination of the molecular make-up, and by extension a more precise characterization of the function, of hemocytes from different body compartments. The genome of *Mercenaria mercenaria*, the hard clam, has recently been assembled at a chromosome level and genes were annotated (14) facilitating the ability to classify genes and proteins of hemocytes into functional groups. Molecular differentiation of hemocytes from hemolymph and EPF can elucidate their specialized roles.

The extrapallial space, between the mantle epithelium and the shell, is the site of shell biomineralization (15). The classical theory of biomineralization is that it is controlled by mantle tissue, which secretes organic materials such as shell matrix proteins and inorganic ions creating the EPF. There is now evidence that hemocytes function in this process, specifically in sequestering calcium (Ca^2+^) and carbonate (CO_3_)^2-^, providing a supply of Ca^2+^ which is a major constraint during calcification (4, 9). Mount et al. (4) proposed that a special type of hemocyte contains Ca^2+^ granular contents and calcium carbonate (CaCO_3_) crystals and can function in biomineralization; however, the role of hemocytes in biomineralization is still not well understood. Regulation of pH in the EPF is critical to maintain calcification, and evidence suggests marine invertebrates can elevate pH in this calcifying fluid relative to the seawater and increase CaCO_3_ saturation through ion transport (16–19). Ivanina et al. (20) demonstrated over expression of ion transporters in hemocytes, indicating that hemocytes are involved in ionic regulation to promote calcification in the EPF.

In addition to participating in biomineralization, hemocytes in the EPF perform phagocytosis and produce a range of humoral factors (21, 22), suggesting EPF might serve other biological processes such as defense. The extrapallial space is an active site for microbial colonization and multiple infections affecting bivalves are initiated in this area. This is the case for juvenile oyster disease (JOD) and brown ring disease (BRD), which are bacterial diseases that respectively affect *Crassostrea virginica* (eastern oyster) and *Ruditapes philippinarum* (Manila clam), and are often associated with hemocyte infiltration to mantle surfaces and the EPF, suggesting localized-immune response at the site of infection (8). Despite these preliminary investigations, additional research is needed to allow the identification of molecular pathways that enable hemocytes in the EPF to contribute to biomineralization while still fulfilling their immune functions.

This study used functional assays, transcriptomics, and proteomics to characterize hemolymph and EPF in *M. mercenaria*, and to determine potential specialization with regard to processes such as biomineralization, ion regulation, and immunity.

## Materials and Methods

### Animals

Adult (50-70 mm) *M. mercenaria* were obtained from a commercial source (Frank M. Flowers and Sons Inc., Oyster Bay, NY). Clams were scrubbed to remove debris and epibionts upon arrival. They were maintained in four aerated aquaria (salinity 30 practical salinity units (PSU), Temperature 25°C, pH 7.8) and fed daily with commercial diet (LPB Frozen Shellfish Diet, Reed Mariculture, City, CA, USA). Body fluids were collected five to seven days later.

### EPF and Hemolymph Collection

**Figure 1:**
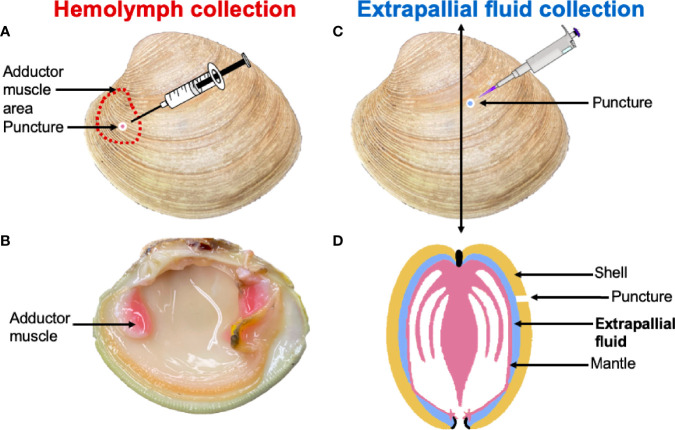
Hemolymph was collected through a puncture drilled over the anterior adductor muscle **(A, B)**. The extrapallial fluid (EPF) was collected through a puncture made in the central part of the shell **(C)**. **(D)** is a schematic representation of a clam cross section that indicates the position of the EPF between the shell and the mantle.

EPF and hemolymph samples were individually collected (**Figure 1**) from 20 clams (five per aquarium). EPF was collected by drilling a hole into the center of the left valve (**Figure 1**) using a round dental bur as described by Allam and Paillard (21). When the EPF volume from one side of the mantle cavity was not sufficient for all downstream analyses (< 1 ml), EPF was collected from both valves of each clam and pooled. A second hole was drilled above the anterior adductor muscle and hemolymph was withdrawn using a syringe. Approximately 1-1.5 ml of each fluid type was collected, and all samples were held on ice. Quality of the EPF was checked by examination of mantle integrity after opening the valves, if the mantle was compromised, a new clam was processed.

Each fluid type was aliquoted, and 100 µl was saved for functional assays and diluted 1:4 with ice cold filtered artificial seawater (FASW 30 PSU). The remaining fluid was centrifuged (800g, 4°C, 10 min). The supernatant was transferred to a new collection tube and protease inhibitor cocktail (SIGMA*FAST*™ Protease Inhibitor Tablets; 50 µl/m1) was added. The pelleted cells and tubes containing supernatant were flash frozen and stored at -80°C.

### Functional Assays

Flow cytometry (BD FACSCalibur) was used to assess hemocyte activities as described below. Most of these assays rely on the assessment of fluorescent signals emitted by hemocytes following the addition of dyes that target specific pathways or molecules. For all these assays, a minimum of 1,000 hemocytes were assessed, and data was compared between EPF and hemolymph using nested ANOVAs. Fluid type was the main effect and the aquarium was the random or nested factor. Data was generated from a total of 20 clams, with five individual clams sampled from four replicate tanks (n=4). Before analyses, cells were separated from debris by size and intracellular complexity, which is a standard procedure for bivalves that does not require the addition of specific dyes that could otherwise alter cellular activities (11, 23).

#### Cell counts and viability

Propidium iodide (PI; ThermoFisher) was added at the final concentration of 20 μg/ml, incubated for 10 min in the dark at room temperature (RT) before flow cytometry readings. PI cannot enter viable cells, but binds to DNA in dead cells, consequently making dead cells more fluorescent in the orange (FL2) channel. Agranulocytes and granulocytes were separated based on light forward (FSC) and side (SSC) scatter parameters and treated separately for the calculation of the percent of dead cells in each hemocyte subpopulation (11).

#### Intracellular pH

Fluid samples were transferred to sealed 0.5 ml microcentrifuge tubes to minimize gas exchange and then immediately centrifuged (800g, 4°C, 10 min). The supernatant was removed and the pellet was resuspended in FASW containing 2′,7′-bis-(2-carboxyethyl)-5-(and-6)-carboxyfluorescein, acetoxymethyl ester (BCECF-AM; Sigma) at a final concentration of 1μM. Samples were incubated at RT in the dark for 10 min before reading on the flow cytometer.

#### Ca^+2^ measurements

Relative Ca^+2^ contents in hemocytes were assessed using Fluo-3 (ThermoFisher), a dye that shows increase in green fluorescence (FL1 channel) intensity with increasing levels of Ca^+2^. Fluo-3 was added at a final concentration of 5 μM and incubated at RT in the dark for 20 min before sample reading.

#### Phagocytosis

Yellow-green latex beads (2 μm; Sigma) were added to samples (1:10 hemocyte:bead ratio) and incubated at RT for 1 hr before sample reading. Hemocytes associated with beads were considered phagocytic.

### RNAseq Library Preparation, Sequencing, and Analysis

RNA was extracted using TRIzol Reagent (Invitrogen, ThermoFisher). DNA was removed using DNA-free™ Kit (Ambion), following manufacturer’s instructions. After checking the quality and quantity of RNA (Nanodrop), samples derived from EPF and hemolymph from the same eight clams were selected for sequencing. Extracted RNA was sent for sequencing to Novogene Corporation (UC Davis, Sacramento, California). One µg RNA per sample was used as input material. Sequencing libraries were generated using NEBNext^®^ Ultra™ RNA Library Prep Kit for Illumina^®^ (NEB, USA), following manufacturer’s instructions and with indices added for demultiplexing of samples. Libraries were sequenced on Illumina platform (Novaseq 6000) and 150 paired end (PE) reads were generated. Novogene performed quality control tests, and cleaned reads were used in downstream analyses. RNASequencing of the EPF generated 351,049,084 PE reads (41,512,382 - 47,766,790/library) and 355,059,084 PE reads for hemolymph (41,115,505-48,985,418/library). Cleaned sequence reads were trimmed based on quality scores (limit 0.05), ambiguous nucleotides (max 2 nucleotides per sequence), and adapters (CLC workbench [version 11.0.1 (https://digitalinsights.qiagen.com)]). Reads were mapped on the predicted genes annotated on the *M. mercenaria* genome (14) using CLC workbench. Bam files were sorted and indexed, and read counts were generated using Samtools idxstats (24) on Stony Brook University’s high-performance computing cluster SeaWulf. Raw read counts were analyzed with R (3.6.3) and the package *DESeq2* from Bioconductor (25) to perform differential gene expression analysis. Significant differences in gene expression between EPF and hemolymph were identified with a cut-off threshold of adjusted p-value <0.05 after Benjamini-Hochberg correction for multiple comparisons and log2 fold change > |2|. Principal component analysis was performed to visualize clustering of samples, a volcano map was created to visualize fold change (**Figure S1**), and heat map was generated for the top 100 differentially expressed genes (DEGs) between EPF and hemolymph (**Figure S2**). Genes translated into proteins (26) were aligned to the KEGG database using BLAST with a minimum e-value of 10^-5^, keeping the best match per protein with a score greater than 90% identity. Functional annotation (domain identification and three best matches on NR were from (14). Gene Ontology (GO) annotation was performed and a GO enrichment analysis of DEGs was done using the *topGO* R package (27).

### Proteomics

Cell-free hemolymph and EPF were solubilized in 5% SDS, 100mM TEAB, 10mM DTT at 55°C for 30 min. Reduced cysteines were alkylated with 20mM iodoacetamide for 30 min at RT in the dark and proteins were acidified with phosphoric acid. Then proteins were micro precipitated with 90% methanol, 50mM TEAB, and bound to S-Trap solid phase cartridges as described elsewhere (28). Protein precipitates were washed with 90% methanol, 50mM TEAB and digested with trypsin at 47°C for two hours before elution with sequential 50mM TEAB, 0.2% formic acid and 50% acetonitrile (ACN), 0.2% formic acid elution steps by centrifugation (4000g 1 min) each.

Peptides were analyzed by C18 reverse phase LC-MS/MS. HPLC C18 columns were prepared using a P-2000 CO2 laser puller (Sutter Instruments) and silica tubing (100µm ID x 20 cm) and were self-packed with 3u Reprosil resin. Peptides were separated using a flow rate of 300 nl/minute, and a gradient elution step changing from 0.1% formic acid to 40% ACN over 90 minutes, followed 90% ACN wash and re-equilibration steps. Parent peptide mass and collision-induced fragment mass information were collected using an orbital trap (Q-Exactive HF; Thermo) instrument followed by protein database searching using Proteome Discoverer 2.4 (Thermo). Electrospray ionization was achieved using spray voltage of ~2.2 kV. Information-dependent MS and MS-MS acquisitions were made using a 100 ms survey scan (m/z 375 – 1400) at 60,000 resolution, followed typically by ‘top 20’ consecutive second product ion scans at 15,000 resolution. Database searching used Proteome Discoverer 2.4 (Thermo). Peptide and spectra false discovery rates were set to 0.05. Sample normalization was based on total peptide amount. Label free quantitation (LFQ) between samples was performed using intensity based pairwise like-peptide comparisons to generate fold change ratios. Protein abundance was based on summed pairwise peptide abundances and t-tests performed. Shared and modified peptides (oxidated-M, deamidated-NQ, dehydrated-ST) were excluded from quantitation. Protein and RNA datasets were integrated and analyzed using JMP10 (SAS) (**Tables S1, S2**). The resulted proteins were mapped to the predicted proteins annotated from the whole genome (14). Gene Ontology (GO) annotation was performed and a GO enrichment analysis of differentially expressed proteins was done using the *topGO* R package (27).

## Results

### Functional Assays

**Figure 2:**
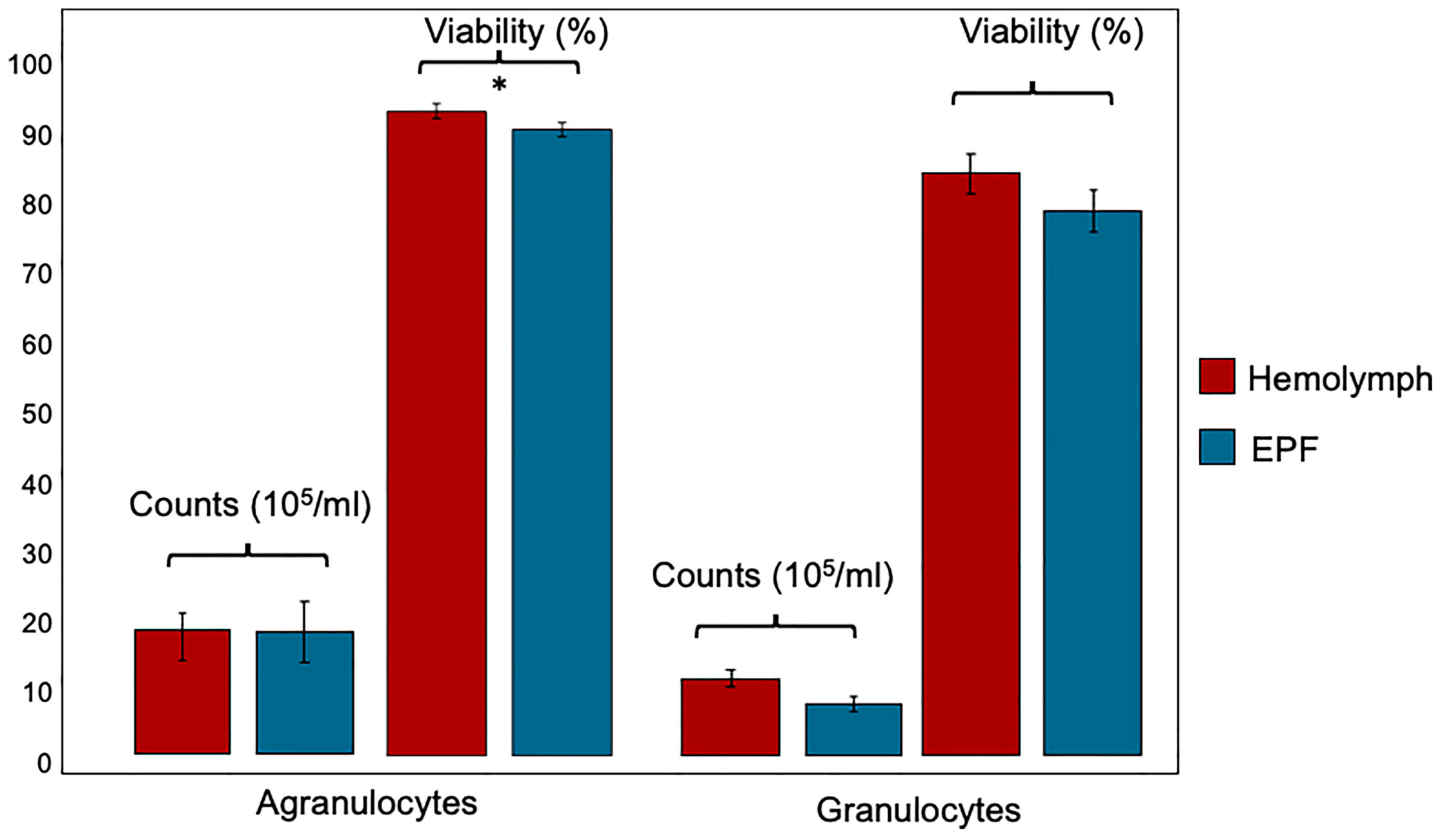
Count and viability (mean ± standard error) of agranulocytes and granulocytes collected from hemolymph and EPF *significant difference between agranulocytes from both fluids (p = 0.022; nested ANOVA; n = 4).

Hemocyte count (nested ANOVA; n=4; p-value=0.46) and the percentage of agranulocytes and granulocytes (nested ANOVA; n=4; p-value=0.73) did not differ significantly between EPF and hemolymph (**Figure 2**). Cell viability was not different between granulocytes collected from both body fluids (nested ANOVA; n=4; p-value 0.14) but agranulocytes were more viable in the hemolymph as compared to EPF (ANOVA; n=4; p-value=0.02) (**Figure 2**).

**Figure 3:**
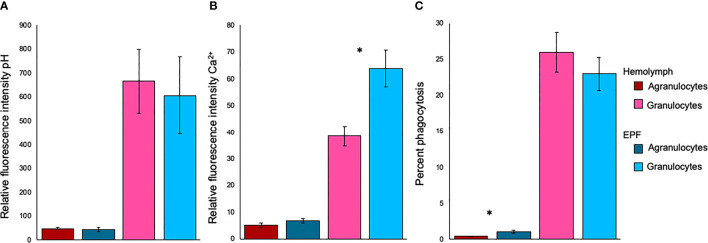
Relative fluorescence intensity of **(A)** BCECF-AM (indicator of intracellular pH) and **(B)** Fluo-3 (indicator of Ca^2+^), and phagocytic activity **(C)** for agranulocytes and granulocytes from EPF and hemolymph. Mean ± standard error. *significantly higher in EPF as compared to hemolymph (p = 0.026 in B and p < 0.001 in C; nested ANOVA; n = 4).

There were no differences in fluorescence intensity after staining with BCECF-AM, suggesting that intracellular pH was similar between agranulocytes (p=0.49) or granulocytes (p=0.51) from both fluids (**Figure 3**). Relative fluorescence intensity of Fluo-3, which is indicative of intracellular Ca^2+^ contents, was significantly higher in granulocytes from the EPF as compared to hemolymph (nested ANOVA; n=4; p-value=0.03) while there were no differences for agranulocytes (nested ANOVA; n=4; p-value=0.14) (**Figure 3**). The percentage of phagocytic agranulocytes was higher in EPF than hemolymph (p<0.001; nested ANOVA; n=4) but there were no significant differences for granulocytes (p-value=0.40) (**Figure 3**).

### Differential mRNA Expression

**Figure 4:**
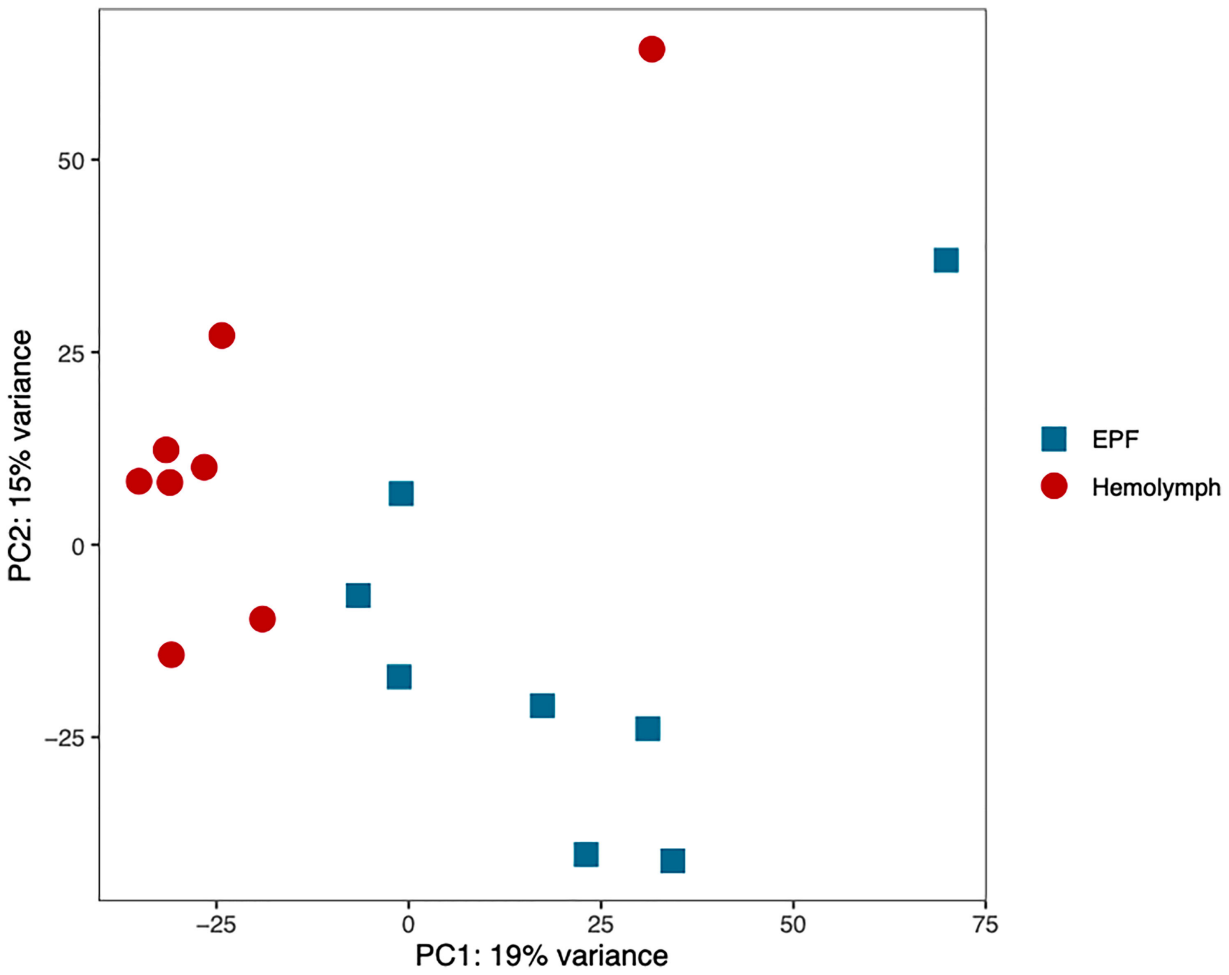
Principal component analysis of the normalized RNAseq data generated from hemocytes collected from the EPF and hemolymph.

Gene transcription profiles were analyzed for hemocytes collected from the EPF and hemolymph. Principal component analysis (PCA) showed separation in gene expression between both fluids (**Figure 4**). 190 genes were differentially expressed, with 113 of these displaying higher expression in EPF as compared to hemolymph (**Table S3**). Interestingly, the PCA showed two outlier samples (one for EPF and one for hemolymph; **Figure 4**) that both originated from the same clam. Since that clam did not display any other unusual conditions (particularly from flow cytometry data), the “outlier” data were kept to ensure that our analysis is conservative and highlights only most significantly different genes.

**Figure 5:**
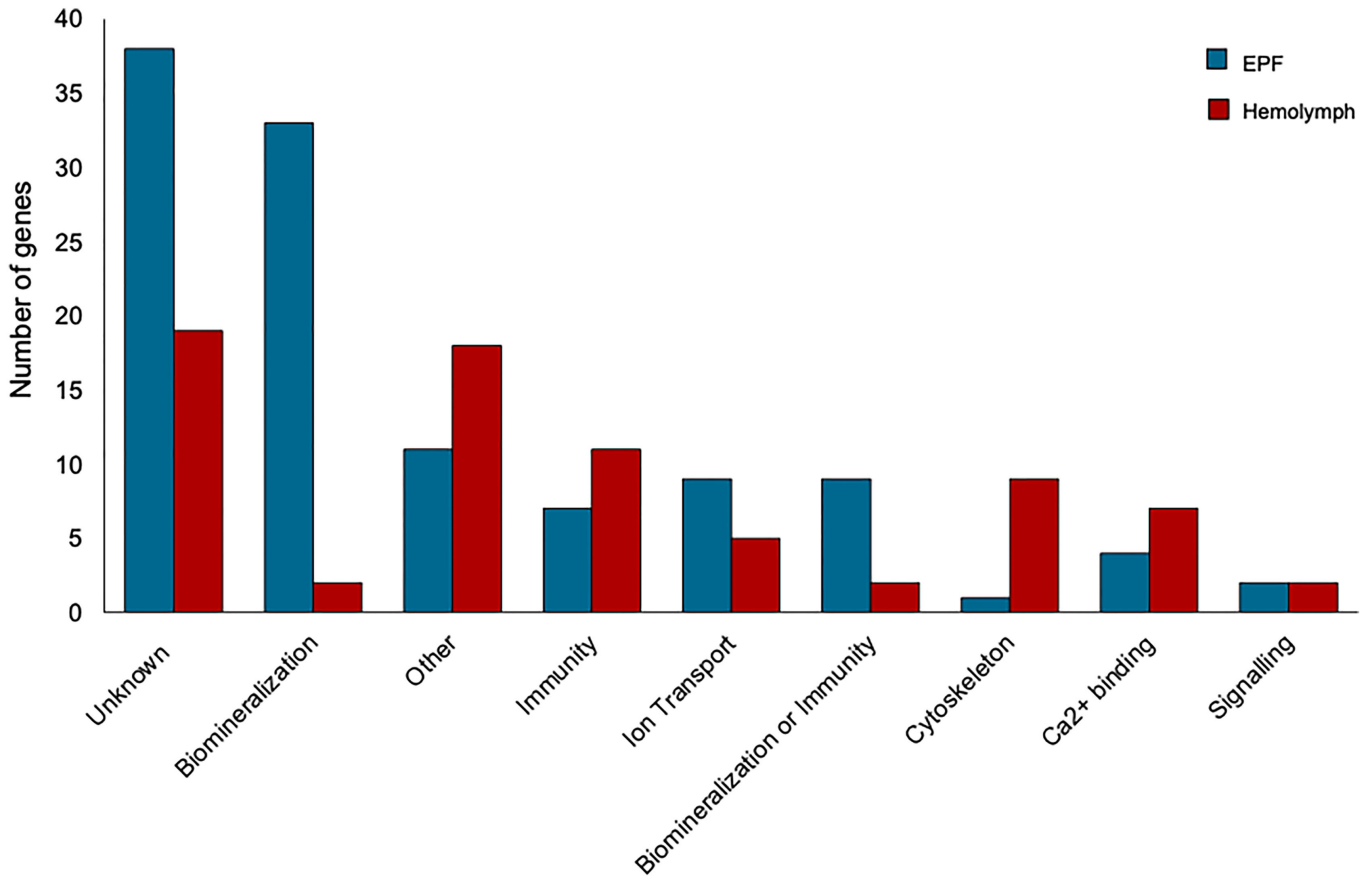
Transcripts overexpressed in hemocytes from the EPF (blue) and hemolymph (red) grouped by their putative functions.

**Figure 6:**
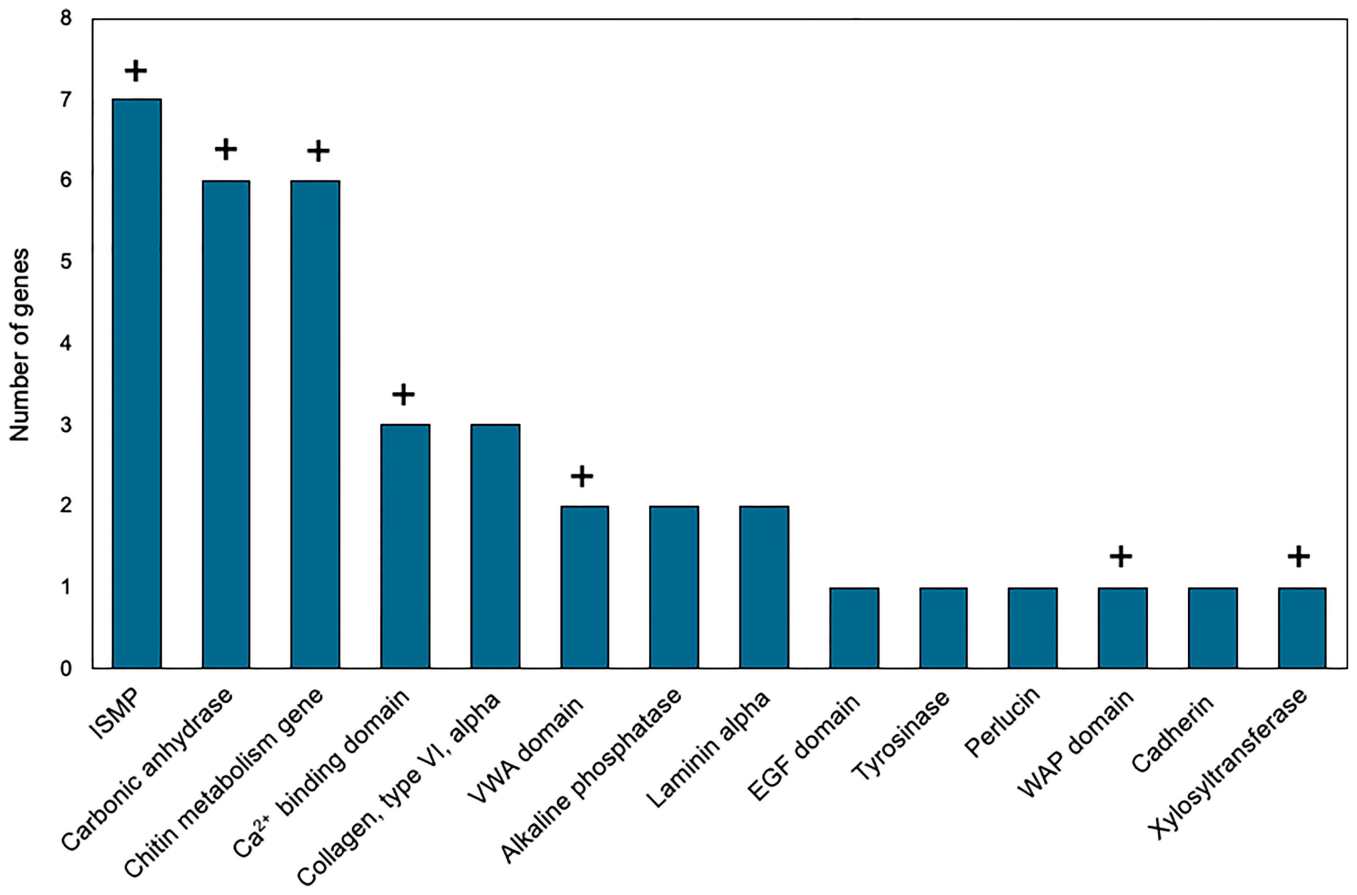
Biomineralization-related genes overexpressed in EPF hemocytes at the mRNA level. ISMP, insoluble shell matrix proteins; VWA, Von Willebrand factor-A; EGF, epidermal growth factor; WAP, Whey Acidic protein; domain names represent genes containing those domains; “+” above the bar indicates that the corresponding genes were also over expressed in the proteome of the EPF plasma (see below).

Genes with higher expression in EPF were surveyed for their associated functions. There were 37 genes overexpressed in EPF associated with biomineralization (**Figures 5**, **6**). These included six carbonic anhydrase genes (CAs), six chitin metabolism genes, tyrosinase, and two genes with Von Willebrand factor-A (VWA) domains which all make up the “basic tool kit” for molluscan biomineralization (29). Other important genes for biomineralization that were overexpressed in EPF included seven insoluble shell matrix proteins (ISMPs), perlucin, a gene with Whey Acidic Protein (WAP) domain, a gene with epidermal growth factor (EGF) domain, four genes with Ca^2+^ binding domains, three collagen, type VI, alpha genes, two alkaline phosphatase genes, two laminin alpha genes, and a xylosyltransferase. Genes with EF-hand calcium-binding domain, EGF-like domain, and VWA domain made up the genes contributing most to the first and second principal component (**Figure S3**). Genes related to transport were also overexpressed in EPF including three ion transporters, four solute carriers, and two bicarbonate exchange genes. While some domains have been traditionally associated with immune functioning, they also are known to function in biomineralization. There were nine genes that could be important for biomineralization and/or immunity. For example, hemocytes collected from EPF showed an overexpression of genes with C1q domain, proteases, and protease inhibitors, including genes with kazal domains and metalloprotease domains, which might function in immunity or biomineralization. Seven genes were related to immune processes including lysozyme, a gene with Ig-like domain profile, a gene with immunoglobulin domain, and a cell death abnormality protein. While not as common, remaining genes had functions related to signaling, RNA processing, metal ion binding, cytoskeleton, digestion, and oxidoreductase activity. Thirty-eight genes did not have a known function. **Figure S4A** shows GO terms enriched in EPF transcriptome including Ca^2+^ binding, chitin metabolic processes, transporter activity, and ion transporter activity.

Seventy-seven genes had higher expression in hemolymph as compared to EPF (**Figure 5**). Of these, 11 genes were associated with immune response including two universal stress proteins, a gene with tumor necrosis factor (TNF) domain, a vitelline membrane outer layer protein 1, three peroxidase genes, macroglobulin, autocrine proliferation repressor protein A-like, and claudin (**Figure 5**). Genes related to cytoskeleton were the next most prevalent group, with nine genes including two tubulin beta genes, two myosin regulatory light chain genes, a microtubule-associated protein futsch-like isoform, two myosin heavy chain genes, and two calmodulin genes. Overexpressed transcripts also included seven genes with Ca^2+^ binding domains and Ca^2+^ ion binding functions. Other notable functional groups included transport, signaling, and protein binding. Finally, there were two genes that might be important to biomineralization or immunity including genes with C1q domains, and two genes linked to biomineralization. Nineteen genes overexpressed in hemolymph did not have known function. **Figure S4B** shows GO terms enriched in the hemolymph transcriptome including Ca^2+^ binding, binding, cytoskeleton, response to stress, and response to oxidative stress.

### Proteomics

10,383 peptides were identified corresponding to 1,602 proteins (**Table S1**). Protein expression was compared between EPF and hemolymph and filtered for log2foldchange > |0.8| resulting in 284 proteins with significantly greater abundance in EPF and 329 higher in hemolymph (**Table S2**).

**Figure 7:**
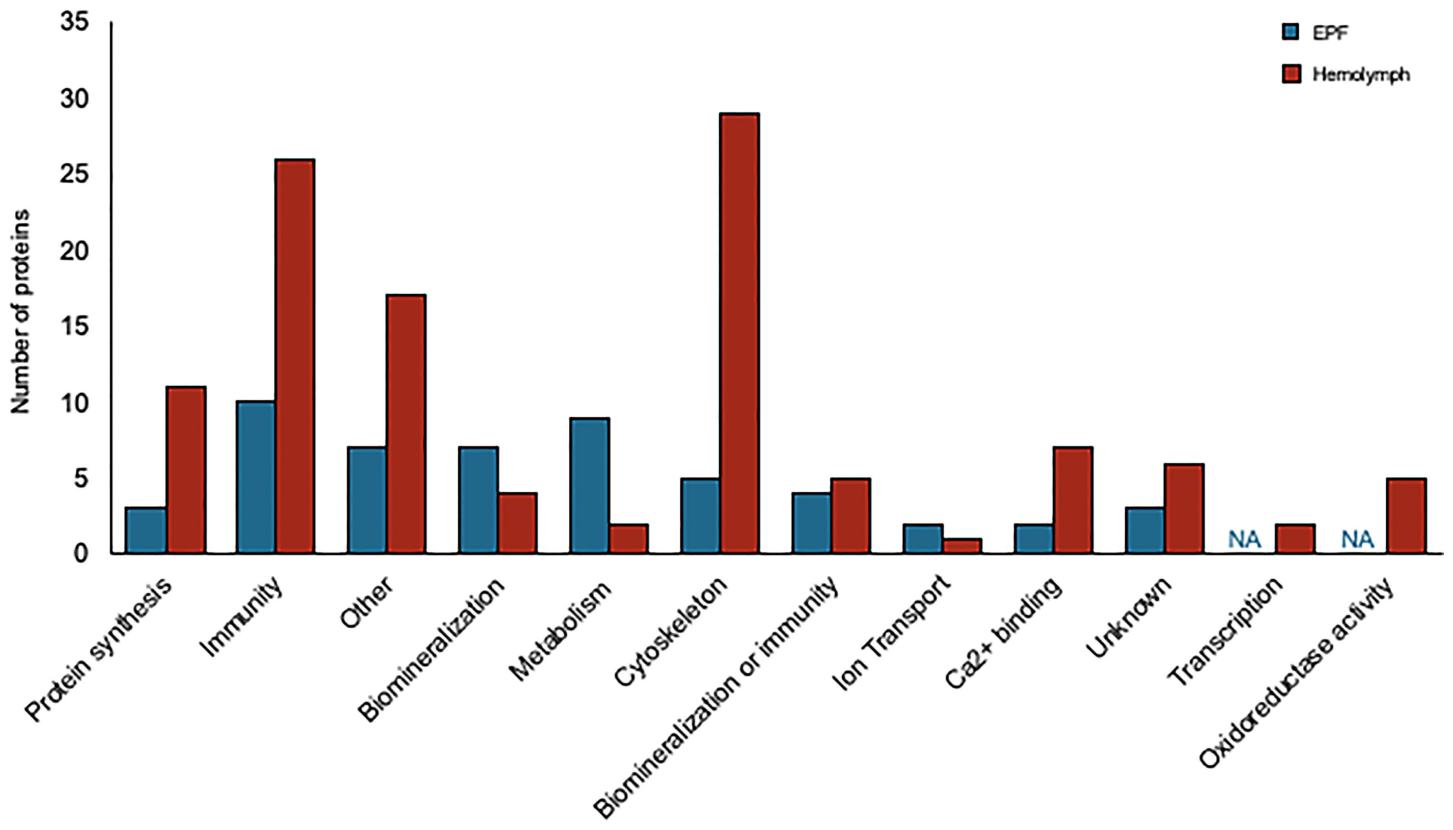
Proteins overexpressed in EPF (blue) and hemolymph (red) plasma grouped by their putative functions.

Convergence between proteomic and transcriptomic results was noted. 171 genes had higher expression in EPF as compared to hemolymph at both the protein and mRNA levels (**Table 1**, **Table S2** and **Figure 7**), or had positive fold change by RNASeq and were significant at the protein level (**Table 1**, **Table S2** and **Figure S5A**). Proteins were also filtered to include those with more than one peptide matching to a protein.

**Table 1:** Representative genes displaying higher expression in the EPF proteome as compared to hemolymph.

ID	Description	Gene LFC	Protein LFC	Functional Group
mRNA.chromosome_10.203.1	WAP domain	6.5	0.87	Biomineralization
mRNA.chromosome_15.1545.1	VWA domain	5.4	1.16	Biomineralization
mRNA.chromosome_6.686.2	CA	3.05	1.24	Biomineralization
mRNA.chromosome_ 1.3030.1	CA	5.69	3.32	Biomineralization
mRNA.chromosome_18.1170.1	ISMP	9.94	1.61	Biomineralization
mRNA.chromosome_18.1174.1	ISMP	7.64	2.72	Biomineralization
mRNA.chromosome_8.1704.1	Xylosyltransferase	2.81	2.87	Biomineralization
mRNA.chromosome_2.2742.1	EF-hand calcium-binding domain	4.48	2.4	Biomineralization
mRNA.chromosome_12.552.1	Chitinase	0.4	1.2	Biomineralization
mRNA.chromosome_9.114.1	Kazal domain	8.96	0.94	Biomineralization/Immunity
mRNA.chromosome_4.844.1	Kazal domain	6.71	3.32	Biomineralization/Immunity
mRNA.contig_2790.1.1	C1q domain	0.76	1.76	Biomineralization/Immunity
mRNA.chromosome_4.347.1	C1q domain	0.4	1.00	Biomineralization/Immunity
mRNA.chromosome_15.1559.1	Trypsin-7	0.4	0.98	Biomineralization/Immunity
mRNA.chromosome_5.362.1	CD109 antigen	6.54	1.01	Biomineralization/Immunity
mRNA.chromosome_13.323.1	Lysozyme C	7.34	2.22	Immunity
mRNA.chromosome_3.1492.1	Complement C1q tumor necrosis factor-related protein 4-like	5.42	1.23	Immunity
mRNA.chromosome_15.1205.2	70 kDa neurofilament protein-like isoform X1	2.42	1.88	Immunity
mRNA.chromosome_9.485.1	Stress-70 protein	1.08	3.32	Immunity
mRNA.chromosome_16.1096.1	Ficolin-1-like	0.04	3.32	Immunity
mRNA.chromosome_19.2906.1	Ficolin-2-like	0.73	0.83	Immunity
mRNA.chromosome_18.298.4	Ig-like domain	0.26	3.32	Immunity

Log2 fold change at mRNA and protein level is provided.

Proteins related to chitinase and CA as well as proteins with Ca^2+^ binding domains and C1q domains were overexpressed in the hemolymph proteome as compared to EPF (**Table 2**). Proteins related to cytoskeleton were particularly overexpressed in the hemolymph proteome, specifically actin-myosin cytoskeleton (**Table S2, Figure S5B**). There were also several immune proteins including heat shock proteins, toll-like receptors, tumor necrosis factor, defensin, lysozyme, galectin, cathepsin, and large subunit GTPase 1 (**Table S2**).

**Table 2:** Representative genes displaying higher expression in the hemolymph proteome as compared to EPF.

ID	Description	Gene LFC	Protein LFC	Functional Group
mRNA.chromosome_9.1851.1	Chitinase	4.46	1.02	Biomineralization
mRNA.chromosome_6.1443.1	Carbonic anhydrase	0.44	0.91	Biomineralization
mRNA.chromosome_11.1876.1	Sarcoplasmic calcium-binding protein	4.03	1.10	Ca^2+^ binding
mRNA.chromosome_2.1676.1	Calcineurin-binding protein	4.59	1.8	Ca^2+^ binding
mRNA.chromosome_13.726.1	Sodium/calcium exchanger regulatory protein 1-like, RS-rich protein-1	5.47	1.47	Ca^2+^ binding
mRNA.chromosome_16.1880.1	C1q domain	1.01	0.91	Biomineralization/Immunity
mRNA.chromosome_12.764.1	C1q domain	0.87	2.06	Biomineralization/Immunity
mRNA.chromosome_16.407.1	C1q domain	0.13	0.93	Biomineralization/Immunity
mRNA.chromosome_18.740.1	C1q domain	0.52	0.86	Biomineralization/Immunity
mRNA.chromosome_10.106.1	C1q domain	0.41	0.84	Biomineralization/Immunity

Log2 fold change at mRNA and protein level is provided.

## Discussion

Prior investigations of hemocytes in bivalves focused on circulating hemocytes in the hemolymph and their role in immunity, overlooking hemocytes in other body fluids such as EPF. Recently, studies have begun to address this gap in knowledge; however, the approaches have mainly focused on morphological and physiological differences between hemocyte groups (22, 30–32) and have not applied -omic tools. This study used functional assays, transcriptomics, and proteomics to further reveal major functions of hemocytes from different fluids and found evidence to support the role of EPF hemocytes in both immunity and biomineralization. In addition, EPF hemocytes appeared to function in ion transport, which could be important for buffering the impact of altered environmental conditions, such as ocean acidification (33, 34). While hemocytes present in hemolymph primarily expressed genes important for immune response and cytoskeleton, there was evidence to suggest hemocytes also contribute to biomineralization with specific genes putatively involved in shell formation, specifically Ca^2+^ binding and transport.

Although evidence suggests hemocytes from hemolymph play a role in shell formation, this study highlights the importance of the EPF in biomineralization, with convergence of evidence from all analyses. The majority of genes with higher mRNA and protein expression in the EPF compared to hemolymph were related to shell formation. Arivalagan et al. (29) proposed a basic tool kit that is required for shell formation. This “tool kit” consists of proteins with the functional domains tyrosinase, CA, chitin-binding-2, and VWA (29). In addition to the proteins explicitly included in the basic toolkit, Arivalagan et al. (29) identified additional proteins that are important for the nucleation and arrangement of CaCO_3_ polymorphs found in the shell. These included EGF and WAP domain-containing proteins (29). In this study, genes with higher mRNA expression in EPF included CA, chitin metabolism and binding genes, tyrosinase, and genes with VWA, EGF and WAP domains. Interestingly, genes with WAP and VWA domains, CA, and chitinase were significantly enriched at both the mRNA and protein level. While not expressly listed in the basic tool kit, other important biomineralization genes were also found overexpressed at both the mRNA and protein level in EPF. For instance, ISMPs were significantly higher in both the transcriptome and proteome of EPF. Insoluble shell matrix proteins (ISMPs), including chitin, create a framework guiding shell formation (35). Xylosyltransferase was also significantly higher at both mRNA and protein level. This gene functions in proteoglycan synthesis, and proteoglycans are involved in CaCO_3_ based biomineralization (36). Similarly, perlucin functions in nucleation of CaCO_3_ crystals [(37); Schwaner, Pales Espinosa, Allam, unpublished), and our results showed this gene to be significantly overexpressed (by RNASeq) and displayed higher relative spectral counts (proteomics, although not significantly overexpressed] in EPF as compared to hemolymph.

It should be noted that the hemolymph also contained genes related to biomineralization that were overexpressed at the mRNA and protein levels, including chitinase and at the protein level CA. Previous studies have shown that hemocytes migrate into the EPF from other areas of the body (9, 38) during infection (39) and shell damage (40, 41). Hemocytes can transport calcite crystals to the site of calcification and are involved in biomineralization (4). Chitin has been suggested to be directly involved in crystal packing and orientation for creation of the foliated microstructure of the inner shell layer, suggesting chitin plays an important role in shell formation (42). Studies have shown that *C. gigas* (Pacific oyster) hemocytes transport Ca^2+^ ions after induced shell damage (41). As mentioned before, a CA was also higher in hemolymph as compared to EPF and it is believed that CAs can play a role in regulating intracellular Ca^2+^ content of hemocytes (43). This supports the idea that hemocytes transport Ca^2+^ to the area of calcification and might be further involved in biomineralization.

During biomineralization, mantle tissue regulates Ca^2+^ turnover, necessitating active ion transport between cells. Ion transporters, bicarbonate transporters, and genes related to ion channel activity are all important for biomineralization. Bicarbonate transporters can supply dissolved inorganic carbon at the site of calcification and regulate pH (44). Yarra et al. (45) investigated the transcriptomes of *Pecten maximus* (great scallop), *C. gigas*, and *Mytulis edulis* (blue mussel) during shell repair and proposed the upregulation of bicarbonate transporters was to regulate the availability of bicarbonate ions to the site of calcification. Genes overexpressed in the EPF transcriptome that could potentially be involved in calcification by transporting ions included ion transporters, solute carriers, and bicarbonate exchange genes. Similarly, ion transporters, solute carriers, and Ca^2+^ binding domains were overexpressed in the EPF proteome. Ca^2+^ content was higher in granulocytes from the EPF than hemolymph, which supports the idea that hemocytes serve as a source of Ca^2+^ during biomineralization. In addition to transporting Ca^2+^ to the site of calcification, some of the aforementioned genes/proteins might be important for acid-base regulation. CA was included in the “tool kit” for biomineralization, because of its role in concentrating inorganic carbon in shell fluid, but it is also very important in acid-base regulation (46). However, there were no differences in intracellular pH between hemocytes from EPF and hemolymph. Similar pH was reported between EPF and hemolymph in *R. philippinarum* (47) and *M. edulis* (48, 49). Instead of raising pH at the site of mineralization to achieve a microenvironment super saturated with respect to carbonate, the overexpression of ion transporters in EPF might be primarily functioning in transportation of Ca^2+^ or CO_3_^2-^. Granulocytes had higher intracellular pH and Ca^2+^ content than agranulocytes, further supporting the role of granulocytes as the main hemocyte subpopulation involved in biomineralization.

Like CA, some genes/proteins can have multiple functions. For example, Arivalagan et al. (29) found immune associated domains in the shell formation proteins. This is particularly true for Ca^2+^-dependent lectins and other lectin-like molecules that sequester Ca^2+^ and are involved in pattern recognition. For example, previous studies in clams showed that complement 1q domain- containing (c1qDC) protein and other complement components are overexpressed in the plasma proteome of clams infected with the fungus-like pathogen *Mucochytrium quahogii* [formerly QPX, (50)]. In fact, complement proteins can function as pattern recognition receptors to identify pathogens and can initiate innate immune response (50–52). In particular, the calcium-dependent lectin-like C1qDC proteins display a marked expansion in bivalve mollusks [primarily to enable a tailored immune response to various microbes; (14, 53)] and have been strongly suggested to be involved in biomineralization as their expression increase after shell damage (45). A similar role for C1qDC in biomineralization has also been suggested in zebrafish (54). The novel lectin family domain Fucolectin tachylectin-4 pentraxin-1 was found in the shell of the clam *Mya truncata* and is associated with innate immunity in other organisms (55). Here, galectin, a highly conserved lectin known to be involved in pathogen recognition and immune response of bivalves (56), was overexpressed in the hemolymph proteome. Galectin has also been overexpressed during shell regeneration in bivalves (9). Proteases and protease inhibitors, both associated with immunity, have also been identified as shell matrix proteins (29). Gerdol et al. (57) demonstrated overexpression of proteases in hemocytes during microbial invasions (57), and metalloprotease domain is a conserved disintegrin domain involved in the inflammatory response (29). Disintegrin and metalloprotease with thrombospondin motifs 16 was overexpressed in the EPF compared to hemolymph at the mRNA level and trypsin- serine protease was over expressed at the protein level. Protease inhibitors are involved in the inflammatory response (29); however, protease inhibitors were also identified as potential shell proteins (29) and have been associated with the fibrous organic matrix between aragonite crystals in *Pinctada fucata* (pearl oyster; 58). A serine protease inhibitor with kazal domain and a tissue inhibitor of metalloproteases were overexpressed at the mRNA level and kazal type serine protease inhibitor at the mRNA and protein level in the EPF. These genes could serve genuine bifunctional roles, adding to the growing evidence of the importance of bivalve hemocytes to both immunity and biomineralization.

While some immune related genes may serve dual functions in biomineralization and immune response, there were several additional genes most commonly associated with innate immunity. In the EPF, lysozyme and genes with Ig-like domain profile and immunoglobulin domain were over expressed at both the mRNA and protein levels. Proteins with tumor necrosis factor domains, stress 70 protein, autophagy-related protein, ficolins, and CD9 antigen isoform were significantly higher in the EPF proteome as compared to hemolymph. Previous studies have demonstrated that EPF hemocytes are involved in phagocytosis (9, 22). There were no significant differences in phagocytosis for granulocytes, which are the most common population of hemocytes performing phagocytosis, between EPF and hemolymph in this study. Total hemocyte count and the percentage of granulocytes and agranulocytes did not differ between EPF and hemolymph. Microbes and pathogens have been documented to infiltrate EPF from the seawater (9) and many pathogens of bivalves can disrupt shell formation (59) or lead to shell diseases (60). JOD and BRD are two bacterial diseases that begin in the periostracum at the edge of the mantle, leading to an inflammatory response characterized by an increase in hemocyte counts in the EPF and alterations in hemocyte activity at the infection loci (39). Allam et al. (39) showed differences in transcription profiles between hemocytes in the circulatory system compared to hemocytes from EPF in clams infected with BRD.

It is already well established that hemocytes in hemolymph are main components of immune response in bivalves (3, 6, 61–65). Both the transcriptome and proteome generated in the current study reflected this, with many immune related genes having higher expression at both the mRNA and protein levels in hemolymph as compared to EPF. Many genes and proteins were associated with signaling. Signaling pathways, including pathogen recognition receptors or toll-like receptors, are an important part of the immune response. Heat shock and stress proteins (higher expression in hemolymph) in bivalves play a role in immune response by contributing to signaling (66) and these pathways were overexpressed in hemolymph. Engulfment of foreign particles during phagocytosis is mediated by the cytoskeleton, specifically the actin-myosin contractile system (67). Hemocytes of the Manila clam had increased expression of cytoskeleton related genes during pathogen exposure (68). A transcriptomic analysis of hemocytes in *C. gigas* found granulocytes with higher expression of genes related to regulation of actin cytoskeleton supporting evidence that granulocytes function in phagocytosis and engulfment of pathogens (69).

Interestingly, the proteome of the cell free plasma and EPF largely reflected the gene expression profiles (from RNASeq) in each body fluid. This is not surprising as many previous studies hypothesized or effectively demonstrated that hemocytes actively secrete a broad range of molecules into the surrounding media (1, 6, 8, 70, 71). The classical theory of bivalve biomineralization contributes proteins found in the EPF to secretion by mantle cells; however, this study suggests that hemocytes contribute to this process as well. It should be noted that proteins found in plasma and cell-free EPF included some that are related to cytoskeleton. Similar findings were reported by Leprêtre et al. (70) and (71) who used proteomics methods and demonstrated that hemocytes and plasma of the freshwater zebra mussel (*Dreissena polymorpha*) share many proteins including those related to cytoskeleton, membrane organization, cell motility, cell adhesion, and metabolic processes (70). Furthermore, recent investigations highlight the ability of hemocytes to release constitutive intracellular components into the plasma. A good example for this process is the release of extracellular traps by bivalve hemocytes (72). In fact, functional tubulin and actin filaments are required in extracellular trap formation (73) and myosin and actin have been identified in traps (74). Interestingly, these proteins were higher in the hemolymph proteome as compared to the EPF proteome. Hemolymph proteome, but not EPF, also contained histone proteins, which are the main proteomic constituent of extracellular traps. The relative abundance of these proteins in cell-free plasma might indicate that the etosis pathway may be more prominent in hemolymph as compared to the EPF.

Together, these findings demonstrate functional specialization of hemocytes between EPF and hemolymph. Variation in the expression of biomineralization-related genes and proteins between these fluids, along with differences in calcium content, underline the role of EPF in shell formation. Hemocytes in the hemolymph played an auxiliary role, by transporting Ca^2+^ into the extrapallial cavity. This compartmentalization may allow the use of hemolymph as an important conveyer of elements (e.g. Ca^2+^) towards calcification sites, while the specific hemocyte activities in EPF may create the microenvironment conducive to biomineralization. Future work is required to further clarify the role of hemocytes in biomineralization, such as their relationship with mantle-mediated calcification. Similarly, studies are needed to determine whether hemocytes in EPF represent a specialized fraction of hemocytes commonly present in the circulatory system or if their unique transcriptomic signature results from a “maturation” process that they undergo when they migrate to the extrapallial space. This study underscores the well-established role of circulatory hemocytes in bivalve defense, while also demonstrating that peripheral compartments including the extrapallial cavity contain hemocytes that function in immune protection of their compartment. Functional analysis of genes that might be important for both biomineralization and immunity, such as genes with C1q and kazal domains, should be further investigated to better elucidate whether a dual function in immunity and biomineralization is truly the case for the same genes or whether different members of these diversified gene families play different roles.

## Data Availability Statement 

The original contributions presented in the study are publicly available. RNASeq data can be found at: https://www.ncbi.nlm.nih.gov/sra under accession number SAMN24368625. Proteomic data can be found at: https://massive.ucsd.edu/ProteoSAFe/static/massive.jsp under accession number MSV000088664.

## Author Contributions

EP and BA led the conceptualization and secured the funding. CS contributed to investigation, methodology, formal analysis, and writing the original draft. SF and JH assisted with the generation and processing of transcriptomic and proteomic data, respectively. All authors participated in manuscript revision. All authors contributed to the article and approved the submitted version.

## Funding

This research was supported by New York Sea Grant program (projects R/XG-24 and R/XG-32).

## Conflict of Interest

The authors declare that the research was conducted in the absence of any commercial or financial relationships that could be construed as a potential conflict of interest.

## Publisher’s Note

All claims expressed in this article are solely those of the authors and do not necessarily represent those of their affiliated organizations, or those of the publisher, the editors and the reviewers. Any product that may be evaluated in this article, or claim that may be made by its manufacturer, is not guaranteed or endorsed by the publisher.
