# Supplementary material for: Transcriptomic, Proteomic, and Functional Assays Underline the Dual Role of Extrapallial Hemocytes in Immunity and Biomineralization in the Hard Clam Mercenaria mercenaria
Source: Front Immunol. 2022 Feb 22;13:838530. doi: 10.3389/fimmu.2022.838530 (PMC8902148; doi:10.3389/fimmu.2022.838530)
Supplement: Supplementary file 1 [file DataSheet_1.docx]

Supplementary Material

**Figure S1**. Volcano map of differentially expressed transcripts of hemocytes from the EPF and hemolymph. Red dots indicate differentially expressed genes with log2fold change >|2| and adjusted p-value < 0.05 and blue dots indicate DEGs with p-value < 0.05 but log2fold change <|2|. Black dots represent non-significant genes.

**Figure S2.** Heat map of top 100 DEGs between EPF and hemolymph hemocytes. mRNA expression data was converted into data matrix with each column representing a sample and each row a DEG. Color of each cell is the expression level with green representing highest expression. Heat map has hierarchical clustering.


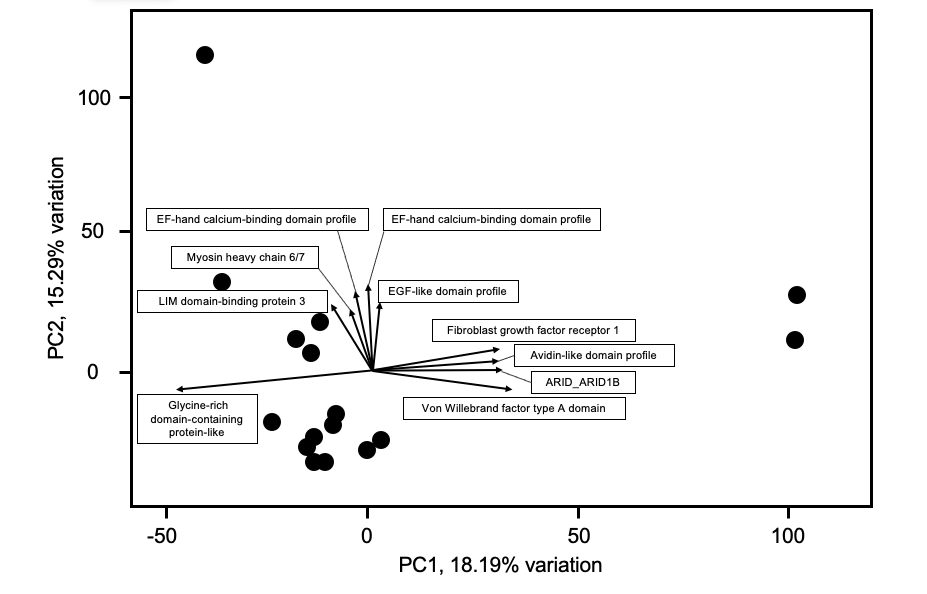


**Figure S3**. Top genes contributing to the first two principal components from the RNAseq data generated from hemocytes collected from the EPF and hemolymph.

**
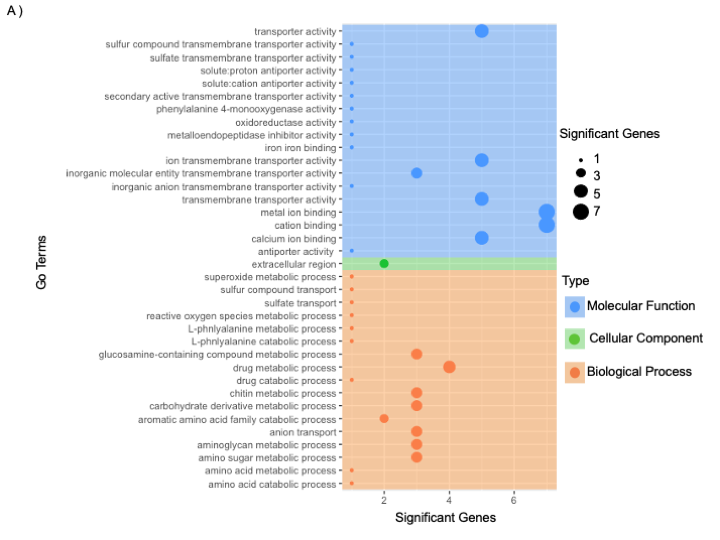
**

**
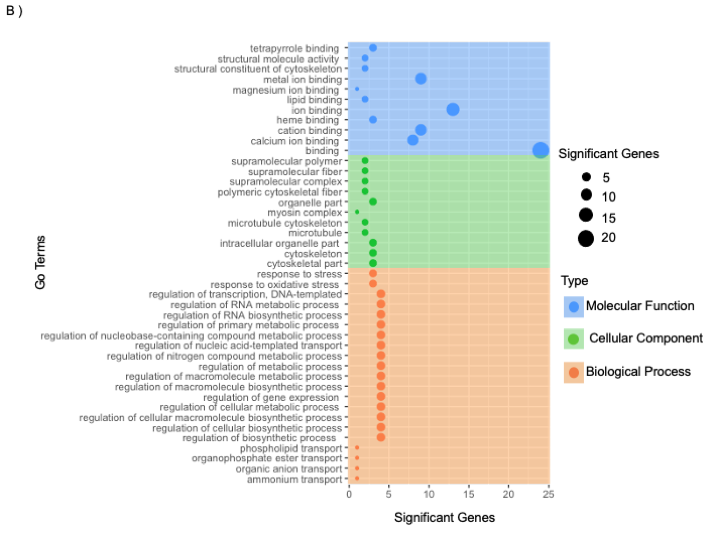
**

**Figure S4**. Number of genes related to GO terms overrepresented in hemocyte transcriptome from (A) EPF and (B) hemolymph. Circle diameter represents number of genes.

**
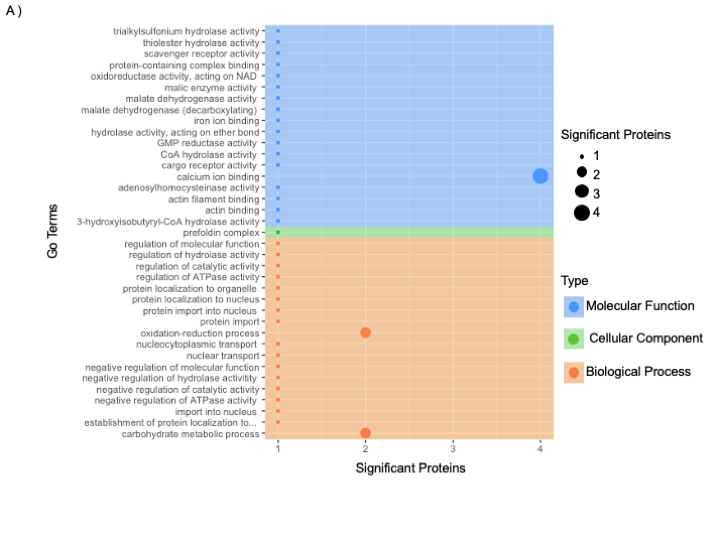
**

**
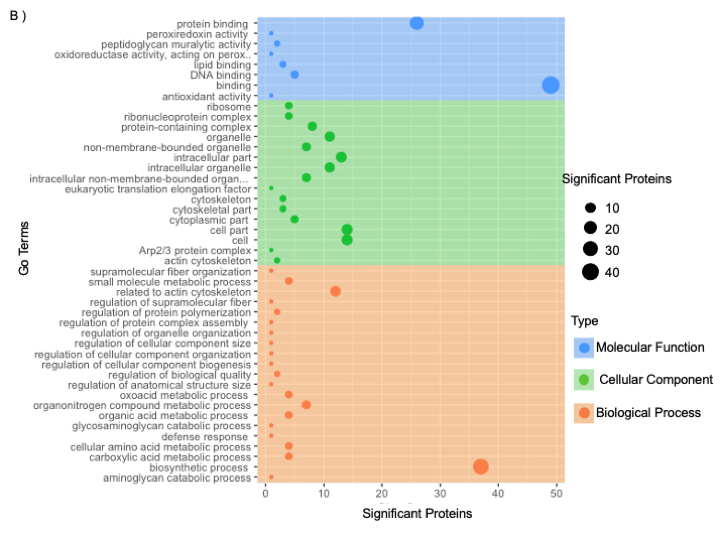
**

**Figure S5**. Number of proteins related to GO terms overrepresented in (A) EPF and (B) hemolymph plasma proteome. Circle diameter represents number of proteins.

**Table S1**. Excel file of protein and mRNA datasets merged

**Table S2**. Merged excel file of protein and mRNA datasets. Sheet 1 proteins with > 0.8 log2foldchange in EPF; sheet 2 filtered for positive fold change in mRNA, and > 1 peptide matching in EPF; sheet 3 proteins with > 0.8 log2foldchange in hemolymph; sheet 4 filtered for positive fold change in mRNA, and > 1 peptide matching in hemolymph

**Table S3**. DEG in the transcriptome with predicted proteins, log2fold change, adjusted p-values, functional groups, associated GO terms and KEGG pathways. Sheet 1 EPF and sheet 2 hemolymph.

**
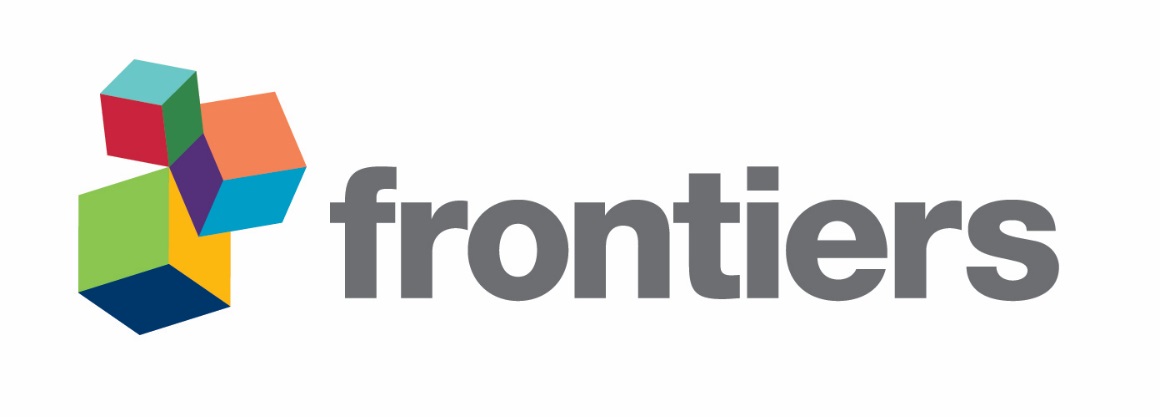
**
